# Evaluation of PPP-ABS investment environment based on combined weighting of level difference maximization and TOPSIS method

**DOI:** 10.1371/journal.pone.0295856

**Published:** 2023-12-14

**Authors:** Lijun Zhang, Junwen Feng, Bo Feng

**Affiliations:** 1 School of Economics and Management, Nanjing University of Science and Technology, Nanjing, Jiangsu, China; 2 School School of Intellectual Property, Nanjing University of Science and Technology, Nanjing, Jiangsu, China; 3 Nanjing Audit University Jinshen College, Nanjing, Jiangsu, China; UCL: University College London, UNITED KINGDOM

## Abstract

Asset-backed securitization (ABS) is currently used to refinance public-private partnership (PPP) projects in the infrastructure field. To stimulate the investors’ enthusiasm, this study evaluated the investment environment of PPP projects asset-backed securitization (PPP-ABS). Firstly, we established a PPP-ABS investment environment evaluation indicator system based on the literature review and the practice of PPP-ABS. Then, the optimal weights of each indicator were determined by the combined weighting of level difference maximization method, where the subjective weights were determined by the AHP method, and the objective weights were determined by the entropy method. Finally, we evaluated the PPP-ABS investment environment from 2015 to 2022 with the technique for order preference by similarity to ideal solution (TOPSIS) method. The final valuation results are consistent with the actual situation. The results showed that the PPP-ABS investment environment exhibits a stable and upward trend. Under the overall guidance of the government, the approval process, information disclosure and supervisory systems have continued to improve, the number of ABS products issued has continued to grow, and the overall market risk is controllable. However, some problems still need to be solved and improved, including inadequate accounting and tax systems, insufficient liquidity in the secondary market, and the recovery of economic development in the post-COVID-19 era. This study fills the research gap in PPP-ABS. It proves the rationality and feasibility of PPP-ABS and is expected to provide a reference for investors’ decision-making and promote the sustainable and healthy development of PPP-ABS.

## 1. Introduction

Infrastructure investment is critical for promoting economic growth and social stability [1–3]. Building sustainable and resilient infrastructure is one of the 17 sustainable development goals(SDGs) proposed by the United Nations [4]. It is also related to the realization of other SGDs. The government encourages the private sector to invest in infrastructure projects in order to reduce financial pressure and improve infrastructure projects’ operation and management efficiency through market competition [5]. Therefore, the PPP model has been proposed and become the main method for the private sector to participate in infrastructure construction [6, 7]. However, with the rapid promotion of PPP, many problems have been exposed, that are not conducive to its sustainable development [8, 9]. ABS can improve the liquidity of basic assets [10, 11], which can activate the existing PPP project assets [12–15]. By the end of 2016, the PPP-ABS was officially proposed in China. PPP-ABS provides opportunities for non-banking financial institutional investors to participate in infrastructure investment and has become an alternative investment and an asset-debt matching tool to achieve long-term capital gains for institutional investors [13]. Individual investors can indirectly invest in PPP-ABS products by purchasing them through financial institutions [16].

Under the guidance of government policies, PPP-ABS has been rapidly promoted and developed. However, PPP-ABS is still in the exploratory stage and investors are mainly commercial banks Existing studies on PPP-ABS have focused only on risk evaluation, product pricing, and feasibility assessment. Existing studies have emphasized the importance of external environmental factors in PPP-ABS, but there is no specific empirical research on the PPP-ABS investment environment from the investors’ perspective. The promotion and sustainable development of PPP-ABS requires active participation and support from investors. The investment environment is a critical external factors that affects investors’ decision-making [17]. Therefore, it is necessary to evaluate the PPP-ABS investment environment.

Evaluation theories and methods are mature. As a commonly used evaluation method, TOPSIS can make full use of the information in the original data and accurately reflect the gap between the evaluation objects. Therefore, it is widely used in multicriteria decision-making (MCDM), and its effectiveness has been proven [18, 19]. Some scholars have adopted the TOPSIS method to evaluate investment environments, and confirmed its applicability and effectiveness [20, 21]. Therefore, this study evaluates the PPP-ABS investment environment with TOPSIS method. In addition, indicator weighting is a key aspect of evaluation. By combining the advantages of subjective and objective weighting methods, this study proposes a combined weighting method. The combined weighting based on level difference maximization has been widely used in evaluation studies in various fields, and its rationality has been confirmed [22–25]. Therefore, this study proposes determining the combined weights of evaluation indicators based on level difference maximization.

This study aims to evaluate the PPP-ABS investment environment based on the combined weighting of level difference maximization and TOPSIS method, with the following practical and academic contributions. Firstly, we established a PPP-ABS investment environment evaluation indicator system based on the practice of PPP-ABS and related literature. Secondly, we proposed to determine the optimal combined weights based on level difference maximization, where the subjective weights were determined by the AHP method, and the objective weights were determined by the entropy method. Finally, we evaluated the PPP-ABS investment environment using the TOPSIS method. This paper provides a systematic evaluation of the PPP-ABS investment environment based on objective data from 2015 to 2022, which fills the research gap in PPP-ABS. The results verify the rationality and feasibility of the PPP-ABS proposal by evaluating the investment environment. It is expected to provide a valuable reference for institutional investors’ decision-making, decrease investment risks, stoke institutional investors’ excitement, and optimize investor structure, all of which are essential for the sustainable and healthy development of PPP-ABS.

The remainder of this paper is organized as follows: In Section 2: Literature review, we analyze the current studies on PPP-ABS and notes their limitations. In Section 3: Research methodology, we describe the research methodology, including the establishment of PPP-ABS investment environment evaluation indicator system, the determination of indicator weights, and the evaluation of PPP-ABS investment environment. In Section 4: Empirical study, the feasibility and effectiveness of the proposed method are verified by an empirical study. In Section 5: Results and discussion, we discuss the results obtained by the proposed method. Finally, conclusions and the future research directions are presented in Section 6:Conclusions.

## 2. Literature review

### 2.1 Related studies of PPP-ABS

Current research on PPP-ABS focuses primarily on risk evaluation, product pricing, and feasibility assessment. All existing studies have confirmed the importance of external environmental factors for PPP-ABS, such as improved legal framework, open issue process, clear regulatory mechanism, relevant tax and accounting treatment standards, transparent information disclosure, mature and effective market, and developed economy.

Chu et al. (2017) collected issued PPP-ABS and infrastructure ABS products data and established a multiple linear regression model to analyze the critical factors affecting the spread pricing of PPP-ABS. Their study found that the year of issuance has a significant impact on the pricing spread of PPP-ABS, indicating that the macroeconomic environment affects product pricing [26]. Lu et al. (2019) identified the critical success factors of PPP-ABS through literature research, case studies, expert interviews, and questionnaires. They derived five dimensions of PPP-ABS assessment framework based on the principal component analysis (PCA), two of which were related to the external environment. Their research highlighted the importance of supporting institutional systems and market factors for PPP-ABS [13]. Liu et al. (2021) determined the critical success factors for PPP-ABS through a questionnaire survey and adopted structural equation modeling to explore the relationship between these factors and the success of PPP-ABS. The results showed that the maturity of relevant institutions, including transparent information disclosure, mature accounting and tax standards, mature secondary market, clear and effective government regulation, will affect the issuance and success of PPP-ABS [12].

Hou (2018) [27], Liu and Gan (2019) [28], Ye and Chen(2020) [29], Zhao et al. (2020, 2021) [14, 30], and Huo and Li(2021) [31] established PPP-ABS risk evaluation indicator systems from different perspectives. All risk evaluation indicator systems established by the above scholars include external environmental factors, including institutions, market, and economy. They determined the importance of these factors using the analytic hierarchy process (AHP) or combined weighting method. Deng(2017) [32], Xing(2017) [33], Han(2017) [16], Zhang(2018) [34], Yu(2018) [35], Tian and Wang(2018) [36], He (2019) [37] emphasized the significance of PPP-ABS and also noted the current problems it faces through qualitative description. Current problems are mainly related to the external environment, such as law, regulation, accounting and tax treatment principles, information disclosure, and other institutions.

### 2.2 Limitations of existing studies

After analyzing the relevant studies, this study found that the research on PPP-ABS has the following limitations:

1. The current PPP-ABS evaluation model is mainly limited to PCA, AHP, fuzzy theory, and Dempster-Shafer (D-S) evidence theory. The determination of indicator weights is subjective. Even if an objective weight method based on information entropy is used, the weight still be calculated based on expert subjective opinion data.
2. Previous studies have emphasized the importance of external environmental factors for PPP-ABS. However, no scholars have yet conducted an evaluation study of the PPP-ABS investment environment from the investors’ perspective.

This study collects actual and objective data and evaluates the PPP-ABS investment environment based on the combined weighting of level difference maximization and TOPSIS method, which can overcome the existing research limitations.

## 3. Research methodology

**Fig 1:**
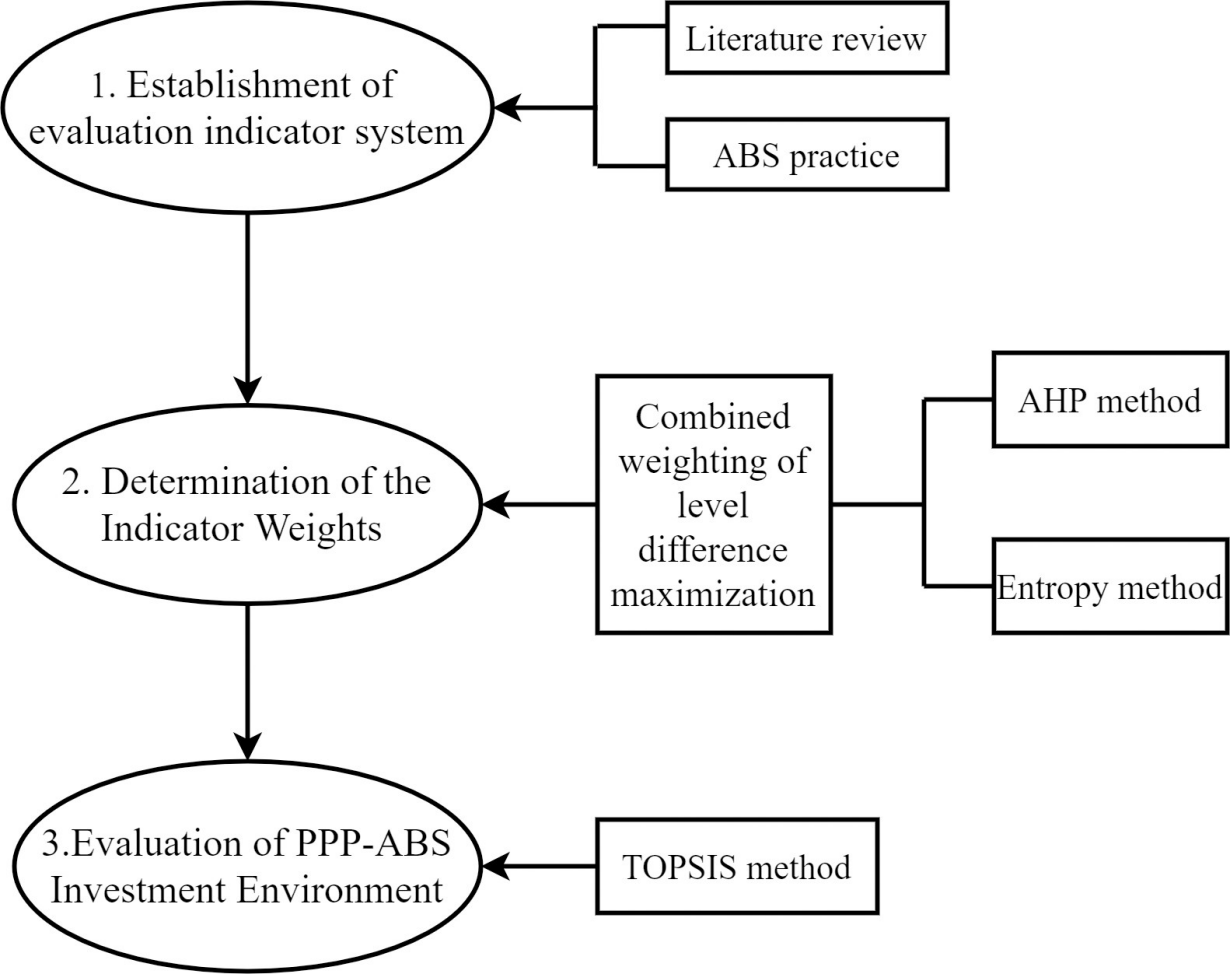
Research framework. This figure illustrates the research process of the evaluation of PPPP-ABS investment environment and the methods used at each step.

This study adopted a literature research approach and combined it with the ABS practice to establish a PPP-ABS investment environment evaluation indicator system. it then determined the indicators weights based on the combined weighting method of level difference maximization, and finally proposed to evaluate the PPP-ABS investment environment using the TOPSIS method. The research framework is shown in Fig 1. The method follows rigorous mathematical analysis and logical reasoning, which made the evaluation results more accurate and scientific.

### 3.1 Establishment of PPP-ABS investment environment evaluation indicator system

The investment environment refers to the macro factors affecting investors’ decision-making, including institutional, market, and economic factors [38]. The investment environment can affect investors’ beliefs and goals. To attract investment, it is necessary to first have a developed economic situation to encourage people to invest, have a good government image and supporting institutional systems to make people willing to invest, and have a mature capital market to allow money to flow. A favorable investment environment positively affects investment decision-making [39]. Based on the literature research and combined with PPP-ABS practice, we established the PPP-ABS investment environment evaluation indicator system from three dimensions: institutional environment, ABS market environment, and economic development environment, as shown in Table 1.

**Table 1:** PPP-ABS investment environment evaluation indicator system.

Target layer	Criterion layer	Indicator layer	Description	Indicator type
Evaluation of PPP-ABS Investment Environment	Institutional environment	Overall guidance	Policies and guidelines	Positive
		Approval process system	The process of issue, transaction, registration and settlement	Positive
		Information disclosure system	Disclosure of investment-related information	Positive
		Tax system	Tax treatment standard for ABS	Positive
		Accounting system	Accounting treatment standards for ABS	Positive
		Supervisory system	Including regulatory body and coordination mechanisms	Positive
	ABS market environment	Issue size	Total annual issuance of ABS products	Positive
		Issue rate	The average issue rate of ABS products	Positive
		Settlement amount	Settlement amount of secondary market	Positive
		Turnover rate	Turnover rate of secondary market	Positive
		Default Rate	Annualized default Rate	Negative
		Early repayment rate	Annualized early repayment rate	Negative
		Rating downgrade Rate	Annualized rating downgrade Rate	Negative
	Economic development environment	GDP growth rate	The national economic growth	Positive
		Income growth rate	The residents’ income level	Positive
		Fixed asset investment growth rate	The situation of fixed asset investment	Positive
		Infrastructure investment growth rate	The situation of infrastructure investment	Positive
		Fiscal revenue growth rate	The situation of government fiscal revenue	Positive
		Fiscal expenditure growth rate	The situation of government fiscal expenditure	Positive

First, the evaluation of PPP-ABS investment environment was divided into three first-level indicators, then they were divided into 19 secondary indicators and explained in detail.

Institutional environment. Government decisions guide investment direction and scale. Favorable policies such as tax incentives and financial subsidies affect investment return rates [40]. The uncertainty of government policies can lead to fluctuations in securities prices, and companies are more inclined to hold more cash and reduce investment in policy-sensitive industries, such as infrastructure [41–44]. An improved institutional environment fundamentally guarantees the project investment returns [45]. The relative institutions of PPP-ABS include overall guidance, approval process system, information disclosure system, tax system, accounting system and supervisory system. This study measures the PPP-ABS institutional environment by the number of documents published by the relevant government departments.

ABS market environment. Research has indicated that the available financial market is a critical factor in infrastructure construction financing through the PPP model [46, 47], and is also essential for the success of PPP-ABS. A mature primary issuance market and an active secondary liquidity market are essential for securities investment [48–50]. We measured the maturity of the primary market by the issue size and rate, and measured the liquidity of the secondary market by the settlement amount and turnover rate. Furthermore, the overall risk in the market can influence investors’ decision-making. Therefore, this study selected the annualized default rate, early repayment rate, and rating downgrade rate to measure the overall market risk.

Economic development environment. Economic fluctuations affect the investment environment. The GDP growth rate is a comprehensive reflection of national economic development. Research shows that GDP growth rate has a significant impact on investment. In addition, the total social investment and the residents’ income level can also affect investment [38]. The underlying asset projects of PPP-ABS belong to the infrastructure field. The growth rate of infrastructure investment reflects the importance the government sector attaches to infrastructure construction, which influences the PPP-ABS investment. The cash flow stability of PPP projects is related to the risk and return of PPP-ABS products. According to statistics, more than 90% of the projects are linked to fiscal revenue and expenditure. Therefore, fiscal situation is an essential factor that cannot be ignored.

### 3.2 Determination of the indicator weights

Indicator weighting is a key factor in evaluating the PPP-ABS investment environment. The weighting methods can be classified into two categories: single weighting method and combined weighting method. The single weighting method is further divided into subjective weighting method and objective weighting method. Subjective weighting methods, such as analytic hierarchy process (AHP) [51], garbage first(G1) [14], and best-worst method (BWM) [52]. determine the indicator weights based on experience and knowledge, which can reflect the decision-maker’s preference but may make the evaluation results too subjective. Objective weighting methods, such as entropy [53, 54], standard deviation [55], and criteria importance though intercrieria correlation (CRITIC) [56], determine the indicator weights based on actual data, but they do not consider the decision-maker’s preferences. The combined weighting method aims to combine the subjective and objective weights based on a specific principle. Commonly used combined weighting methods include additive synthesis, multiplicative synthesis, level difference maximization, and objective modified subjective method. Li et al. (2017) analyzed the rationality of the above four combined weighting methods through an empirical study, and found the combined weighting based on level difference maximization was reasonable [57]. Subsequently, combined weighting based on level difference maximization has been widely used in evaluation studies in various fields [22–25].

Considering the limitations of the subjective and objective weighting methods, this paper proposes the combined weighting method based on level difference maximization. Firstly, the subjective weights were determined by AHP method, and then the objective weights were determine by entropy method. Finally, optimization model were constructed to maximize the variance of the comprehensive evaluation results and to calculate the combined weights.

#### 3.2.1 Subjective weights based on AHP method

The AHP method was proposed by T. L. Saaty in the early 1970s [51, 58]. This method integrates quantitative and qualitative analysis to improve the accuracy of evaluation, and it is widely used in various fields of research [53, 54]. The specific steps of the AHP method are as follows:

Step 1: Construct the judgement matrix. Determine the relative importance between indicators based on Saaty’s 1–9 scale, as shown in Table 2. Then we can obtain the judgement matrix A = (*a*_*ij*_)_n×n_, where *a*_*ij*_ is the element of the judgement matrix, indicating the relative importance of indicator *i* to indicator *j*.

**Table 2:** Saaty’s definition and description of importance scale.

1–9 scale	Definition
**1**	The indicator *i* is as important as indicator *j*
**3**	The indicator *i* is slightly important than the indicator *j*
**5**	The indicator *i* is obviously important than the indicator *j*
**7**	The indicator *i* is strongly important than the indicator *j*
**9**	The indicator *i* is extremely important than the indicator *j*
**2,4,6,8**	Intermediate value of the two adjacent judgments
**Reciprocal**	If the importance ratio of indicator *i* to indicator *j* is *a*_*ij*_, then the importance ratio of indicator *j* to indicator *i* is *a*_*ji*,_ satisfying *a*_*ji*_ *= 1/a*_*ij*_

The scoring standard of the indicators are stipulated.


$$A=[\begin{array}{cccc} a_{11} & a_{12} & \ldots & a_{1n}\\ a_{21} & a_{22} & \ldots & a_{2n}\\ \vdots & \vdots & \vdots & \vdots \\ a_{n1} & a_{n2} & \ldots & a_{nn} \end{array}]$$ (1)

Step 2: Calculate weights. According to Eq (2), we can obtain the normalized judgment matrix $\bar{A}=({\bar{a_{ij}})}_{n\times n}$, where $\bar{a_{ij}}$ is the element of the normalized judgment matrix.


$$\bar{a_{ij}}=\frac{a_{ij}}{\sum_{i=1}^{n}a_{ij}}$$ (2)

According to Equ (3), we can obtain the weight vector $W=\left[\begin{array}{c} w_{1}\\ w_{2}\\ \vdots \\ w_{n} \end{array}\right]$, where *w*_*i*_ is the weight of the *i*th indicator.


$$w_{i}=\frac{\sum_{j=1}^{n}\bar{a_{ij}}}{\sum_{i=1}^{n}\sum_{j=1}^{n}\bar{a_{ij}}}$$ (3)

Step 3: Test consistency. According to Eq (4) to (6), we can obtain the largest eigenvalue *λ*_*max*_, consistency index *CI* and consistency ratio *CR*. *RI* is the average random consistency index, as shown in Table 3. If CR<0.1, the results of the hierarchy analysis are considered consistent, and the determined weights are reasonable.


$$\lambda_{max}=\frac{1}{n}\sum_{i=1}^{n}\frac{{\left(Aw\right)}_{i}}{w_{i}}$$ (4)

$$CI=\frac{\lambda_{max}-n}{n-1}$$ (5)

$$CR=\frac{CI}{RI}$$ (6)

**Table 3:** The average random consistency index RI values.

Matrix Order	1	2	4	5	6	7	8	9	10
**RI**	0	0	0.90	1.12	1.24	1.32	1.41	1.45	1.49

The values of average random consistency index are stipulated.

#### 3.2.2 Objective weights based on entropy method

In 1948, Shannon advanced concept of information entropy based on the theory of thermal entropy in physics and information theory, which is used to describe the average uncertainty between signals [59]. Later, scholars used information entropy to determine the indicator weights. The entropy weighting method aims to determine the objective weight based on the dispersion of indicator data among evaluation objects [54, 60]. The specific steps of the entropy method are as follows:

Step 1: Data collection. The original evaluation matrix *R* as follows:

$$R={\left(r_{ij}\right)}_{m\times n}=[\begin{array}{cccc} r_{11} & r_{12} & \cdots & r_{1n}\\ r_{21} & r_{22} & \cdots & r_{2n}\\ \vdots & \vdots & \ddots & \vdots \\ r_{m1} & r_{m2} & \cdots & r_{mn} \end{array}]$$ (7)

Where $r_{ij}(i=1,2,\cdots ,m;j=1,2,\cdots ,n)$ is the original value of the *i*th evaluation object on the indicator *j* and *m* is the number of evaluation objects, *n* is the number of evaluation indicators.

Step 2: Data normalization. The positive indicators are normalized according to Eq (8), and the negative indicators are normalized according to Eq(9), then we can obtain the normalized evaluation matrix *X*.


$$x_{ij}=\frac{r_{ij}-min(r_{ij})}{max(r_{ij})-min(r_{ij})}$$ (8)

$$x_{ij}=\frac{max(r_{ij})-r_{ij}}{max(r_{ij})-min(r_{ij})}$$ (9)

$$X={\left(x_{ij}\right)}_{m\times n}=[\begin{array}{cccc} x_{11} & x_{12} & \cdots & x_{1n}\\ x_{21} & x_{22} & \cdots & x_{2n}\\ \vdots & \vdots & \ddots & \vdots \\ x_{m1} & x_{m2} & \cdots & x_{mn} \end{array}]$$ (10)

Step 3: Calculation of the information entropy. The information entropy of the *j*th indicator *E*_*j*_ can be calculated as follows:

$$\left\{ \begin{array}{c} E_{j}=\frac{1}{\ln m}\sum_{i=1}^{m}P_{ij}\ln P_{ij}\\ P_{ij}=\frac{x_{ij}}{\sum_{i=1}^{m}x_{ij}} \end{array}\right.$$ (11)

Step 4: Calculation of indicator weights. The weight of the *j*th indicator *w*_*j*_ can be calculated as follows:

$$w_{j}=\frac{1-E_{j}}{\sum_{j=1}^{n}\left(1-E_{j}\right)}$$ (12)

#### 3.2.3 Combined weights based on level difference maximization

The combined weighting method combines the opinions of experts in the AHP subjective weighting method and uses the information entropy of the objective data to avoid subjective error, thereby making the final indicator weights more scientific and reasonable. Determining the combined weights based on level difference maximization can fully reflect the differences between the evaluation objects. This paper constructs the combined weighting optimization model with the goal of maximizing the variance of the comprehensive evaluation results, and the specific steps are as follows:

Step 1: Construct weights matrix *W*. Let $w_{kj}(k=1,2,\cdots ,h;j=1,2,\cdots n)$ demote the weight of the *j*th indicator determined by the *k*th weighting method, then the weight matrix W is:

$$W={{[w}_{jk}]}_{n\times h}=\left[\begin{array}{cccc} w_{11} & w_{12} & \cdots & w_{1n}\\ w_{21} & w_{22} & \cdots & w_{2n}\\ \vdots & \vdots & \ddots & \vdots \\ w_{h1} & w_{h2} & \cdots & w_{hn} \end{array}\right]=\left[\begin{array}{c} W_{1}\\ W_{2}\\ \vdots \\ W_{h} \end{array}\right]$$ (13)

Step 2: Determine the combined weighting principle. Synthesize the advantages of different weighting methods and consider the following combined weighting principle:

$$W_{C}=\delta_{1}W_{1}+\delta_{2}W_{2}+\cdots +{\delta_{h}W}_{h}=\delta W$$ (14)

Where *W*_*C*_ is the un-normalized combined weight vector, *δ*_*k*_(*k* = 1,2,⋯,*h*) is the combined coefficients, and $\sum_{k=1}^{h}{\delta_{k}}^{2}=1$, that is *δδ*^*T*^ = 1.

Step 3: Calculate the comprehensive evaluation results and the variance.

Normalize the original data according to Eqs (8) and (9) to eliminate the influence of the magnitude. The normalized comprehensive evaluation result of the *i*th evaluation object is:

$$Z_{i}=W_{C}X_{i}$$

$$X_{i}={\left[x_{i1},x_{i2},\cdots ,x_{in}\right]}^{T},\;i=1,2,\cdots ,m$$ (15)

Mean value of comprehensive evaluation results is:

$$\bar{Z}=\frac{1}{m}(W_{C}X_{1}+W_{C}X_{2}+\cdots +W_{C}X_{m})$$

$$=\frac{1}{m}W_{C}([X_{1}+X_{2}+\cdots +X_{m}])$$

$$=W_{C}\bar{X}$$ (16)

Let ${X_{i}}^{*}=X_{i}-\bar{X},{{H_{i}=X}_{i}}^{*}({{X_{i}}^{*}}^{T})$, $H=\sum_{i=1}^{m}H_{i}$, then the variance of the comprehensive evaluation value is:

$$S^{2}=\frac{1}{m-1}\sum_{i=1}^{m}{\left(Z_{i}-\bar{Z}\right)}^{2}=\frac{1}{m-1}\sum_{i=1}^{m}{\left[W_{C}(X_{i}-\bar{X})\right]}^{2}$$

$$=\frac{1}{m-1}\sum_{i=1}^{m}{\left({W_{C}X_{i}}^{*}\right)}^{2}=\frac{1}{m-1}\sum_{i=1}^{m}{W_{C}X_{i}}^{*}{\left({{W_{C}X}_{i}}^{*}\right)}^{T}$$

$$=\frac{1}{m-1}\sum_{i=1}^{m}W_{C}H_{i}{W_{C}}^{T}=\frac{m}{m-1}W_{C}H({W_{C})}^{T}$$

$$=\frac{1}{m-1}\delta WH({\theta W)}^{T}=\frac{1}{m-1}\delta WHW^{T}\delta^{T}$$ (17)

Step 4: Construct a combined weighting optimization model and calculate the optimal solution. To fully reflect the overall variability of the evaluation objects, construct combined weighting optimization model with the maximum variance of the comprehensive evaluation value, as follows:

$$max\;F(\delta )=\frac{1}{m-1}\delta WHW^{T\delta^{T}}$$

$$s.t.\left\{ \begin{array}{c} \delta \delta^{T}=1\\ \delta \geq 0 \end{array}\right.$$ (18)

The above optimization problem can be simplified as follows:

$$max\;F(\delta )=\frac{\delta WHW^{T}\delta^{T}}{\delta \delta^{T}}$$ (19)

Where *WHW*^*T*^ is a symmetric matrix, *F*(*δ*) is the Rayleigh quotient of the coefficient vector and there exists a maximum value. Assume *λ*_*max*_ is the largest eigenvalue of the matrix *WHW*^*T*^, *δ* is the corresponding eigenvector [25, 60]. Calculate *δ*, and then obtain the un-normalized combined weights $W_{C}=(w_{c1},w_{c2},\cdots ,w_{cn})$ according to Eq (14). Normalize *W*_*C*_ according to Eq (20) and obtain the normalized optimal combined weights $W_{c}^{*}=(w_{c1}^{*},w_{c2}^{*},\cdots ,w_{cn}^{*})$.


$$w_{cj}^{*}=\frac{w_{cj}}{\sum_{j=1}^{n}w_{cj}}$$ (20)

### 3.3 Evaluation of PPP-ABS investment environment with TOPSIS method

Evaluation theories and methods are mature. Commonly used evaluation methods include fuzzy comprehensive evaluation [61], grey correlation analysis [62], AHP [63, 64], principal component analysis (PCA) [65], TOPSIS [18, 19, 29, 66], and data envelopment analysis (DEA) [67]. Among them, the TOPSIS method proposed by C.L. Hwang and K. Yoon in 1981 [68], makes full use of the information of the original data and accurately reflect the gap between the evaluation objects. Therefore, it hase been widely used in multi-criteria decision-making (MCDM) over the past few decades with satisfactory results [66, 69–72]. Some scholars have adopted the TOPSIS method to evaluate investment environments and confirmed its applicability and effectiveness [20, 21]. Therefore, this study uses the TOPSIS method to evaluate the PPP-ABS investment environment. The TOPSIS method consists of two basic concepts: positive ideal solution and negative ideal solution. This method determines relative superiority of evaluation objects based on the distance from the ideal solutions. The specific steps are as follows:

Step 1: Calculate the standardized score. Assume *y*_*ij*_ is the standardized score of the *i*th evaluation object on the *j*th indicator, which is calculated as follows:

$$y_{ij}=w_{j}*x_{ij}$$ (21)

Where *w*_*j*_ is the weight of the indicator *j* determined by the combined weighting method, and *x*_*ij*_ is the normalized value of the *i*th evaluation object on the *j*th indicator.

Step 2: Determine the positive ideal solution *y*^+^ and negative ideal solution *y*^−^.


$$\left\{\begin{array}{c} y_{j}^{+}=max\;(y_{1j},y_{2j},\cdots ,y_{ij})\\ y_{j}^{-}=min\;(y_{1j},y_{2j},\cdots ,y_{ij}) \end{array},i=(1,2,\cdots ,m\right)$$ (22)

$$\left\{ \begin{array}{c} y^{+}=(y_{1}^{+},y_{2}^{+},\cdots ,y_{n}^{+})\\ y^{-}=(y_{1}^{-},y_{2}^{-},\cdots ,y_{n}^{-}) \end{array}\right.$$ (23)

Step 3: Calculate the distance of each evaluation object to *y*^+^ and *y*^−^.


$$\left\{ \begin{array}{c} d_{i}^{+}=\sqrt{{\left(y_{1}^{+}-y_{i1}\right)}^{2}+{\left(y_{2}^{+}-y_{i2}\right)}^{2}{+\cdots +(y_{n}^{+}-y_{in})}^{2}}\\ d_{i}^{-}=\sqrt{{\left(y_{1}^{-}-y_{i1}\right)}^{2}+{\left(y_{2}^{-}-y_{i2}\right)}^{2}{+\cdots +(y_{n}^{-}-y_{in})}^{2}} \end{array}\right.$$ (24)

Where $d_{i}^{+}$ and $d_{i}^{-}$ are the Euclidean distance of the evaluation object *i* from *y*^+^ and *y*^−^, respectively.

Step 4: Calculate the relative closeness *C*_*i*_.


$$C_{i}=\frac{d_{i}^{-}}{d_{i}^{-}+d_{i}^{+}}$$ (25)

Where *C*_*i*_ is the relative closeness of the evaluation object *i* to the ideal solution, and the greater the value, the better the evaluation effect of the object.

## 4. Empirical study

Existing studies have emphasized the importance of external environmental factors in PPP-ABS, but no specific empirical research on PPP-ABS investment environment from the investors’ perspective has been conducted. To stimulate the investors’ enthusiasm for PPP-ABS, this study evaluated the investment environment from 2015 to 2022 to confirm the rationality and feasibility of PPP-ABS.

### 4.1 Selection of sample data

We established a PPP-ABS investment environment evaluation indicator system in the previous section, and here we select the indicator data for analysis. The number of policy documents published by relevant departments reflects the importance that the government attaches to ABS. Therefore, this study proposes to measure the institutional environment by the number of policy documents published in previous years plus the number of new documents published in the current year. The relevant data were obtained from the websites of the *China the Securities Regulatory Commission* (CSRC), *China Banking and Insurance Regulatory Commission* (CBIRC), *the People’s Bank of China* (PBC), *Shanghai Stock Exchange* (SHSE), and *Shenzhen Stock Exchange* (SZSE). Data about the ABS market environment were obtained from the websites of *China Asset-Backed Securities* (CNABS) and *China Central Depository & Clearing* (CCDC). The secondary market’s settlement amount and turnover rate took credit ABS for example. Data about the economic development environment were obtained from the *National Bureau of Statistics*. The original data for PPP-ABS investment environment evaluation indicators from 2015 to 2022 are shown in Table 4.

**Table 4:** Original data for PPP-ABS investment environment evaluation indicators from 2015 to 2022.

Indicators	Year							
	2015	2016	2017	2018	2019	2020	2021	2022
Overall guidance	15	18	20	24	28	36	40	43
Approval process system	12	13	17	21	21	23	24	28
Information disclosure system	8	9	9	13	15	15	15	15
Tax system	3	3	4	4	4	4	4	4
Accounting system	1	1	1	1	1	1	1	2
Supervisory system	13	14	14	18	26	27	27	27
Issue size (Unit: billion, CNY)	603.24	842.05	1451.98	2012.00	2343.94	2874.93	3099.93	1977.27
Issue rate (%)	4.57	4.05	5.32	5.07	4.17	3.94	3.65	3.03
Settlement amount (Unit: billion, CNY)	39.43	143.53	139.08	248.79	473.04	476.29	1045.71	959.10
Turnover rate (%)	7.44	24.93	15.95	16.81	24	21.83	40.67	40.09
Default rate (%)	0.01	0.02	0.03	0.05	0.06	0.03	0.02	0.03
Early repayment rate (%)	2.35	3.13	1.76	1.86	2.09	1.77	1.64	1.46
Rating downgrade rate (%)	4.83	0.41	1.82	0.51	0.17	0.18	0.01	0
GDP growth rate (%)	7	6.8	6.9	6.7	6	2.2	8.4	3
Income growth rate (%)	7.4	6.3	7.3	6.5	5.8	2.1	8.1	2.9
Fixed asset investment growth rate (%)	8.6	7	6.2	5.9	5.1	2.7	4.9	4.9
Infrastructure investment growth rate (%)	17.2	17.4	19	3.8	3.8	0.9	0.4	9.4
Fiscal revenue growth rate (%)	5.8	4.5	7.4	6.2	3.8	-3.9	10.7	0.6
Fiscal expenditure growth rate (%)	13.2	6.3	7.6	8.7	8.1	2.9	0	6.1

The sample data were selected for the subsequent analysis. The data for each system refer to the number of relevant policy documents.

### 4.2 Determination of indicator weights

Step 1: Determine subjective weights by the AHP method. Invite PPP or ABS experts to score the relative importance of indicators according to the scoring standards shown in Table 2 and obtain a judgement matrix. We can then obtain the AHP weights *W*_*1*_ according to Eq (1) to (6).

W_1_ = [0.0143, 0.0348, 0.0225, 0.0061, 0.0091, 0.0531, 0.0318, 0.053, 0.0901, 0.0901, 0.1537, 0.0212, 0.0901, 0.1122, 0.0165, 0.0264, 0.0396, 0.0693, 0.0693]

Step 2: Determine objective weights by entropy method. The data in Table 6 were first standardized according to Eqs (8) and (9), and the normalized matrix *X* was obtained.


$$X={\left(x_{ij}\right)}_{m\times n}=\left[\begin{array}{llllllll} 0.0000 & 0.1071 & 0.1786 & 0.3214 & 0.4643 & 0.7500 & 0.8929 & 1.0000\\ 0.0000 & 0.0625 & 0.3125 & 0.5625 & 0.5625 & 0.6875 & 0.7500 & 1.0000\\ 0.0000 & 0.1429 & 0.1429 & 0.7143 & 1.0000 & 1.0000 & 1.0000 & 1.0000\\ 0.0000 & 0.0000 & 1.0000 & 1.0000 & 1.0000 & 1.0000 & 1.0000 & 1.0000\\ 0.0000 & 0.0000 & 0.0000 & 0.0000 & 0.0000 & 0.0000 & 0.0000 & 1.0000\\ 0.0000 & 0.0714 & 0.0714 & 0.3571 & 0.9286 & 1.0000 & 1.0000 & 1.0000\\ 0.0000 & 0.0957 & 0.3399 & 0.5642 & 0.6972 & 0.9099 & 1.0000 & 0.5503\\ 0.6725 & 0.4454 & 1.0000 & 0.8908 & 0.4978 & 0.3974 & 0.2707 & 0.0000\\ 0.0000 & 0.1034 & 0.0990 & 0.2081 & 0.4309 & 0.4341 & 1.0000 & 0.9139\\ 0.0000 & 0.5263 & 0.2561 & 0.2820 & 0.4983 & 0.4330 & 1.0000 & 0.9825\\ 1.0000 & 0.8000 & 0.6000 & 0.2000 & 0.0000 & 0.6000 & 0.8000 & 0.6000\\ 0.4671 & 0.0000 & 0.8204 & 0.7605 & 0.6228 & 0.8144 & 0.8922 & 1.0000\\ 0.0000 & 0.9151 & 0.6232 & 0.8944 & 0.9648 & 0.9627 & 0.9979 & 1.0000\\ 0.7742 & 0.7419 & 0.7581 & 0.7258 & 0.6129 & 0.0000 & 1.0000 & 0.1290\\ 0.8833 & 0.7000 & 0.8667 & 0.7333 & 0.6167 & 0.0000 & 1.0000 & 0.1333\\ 1.0000 & 0.7288 & 0.5932 & 0.5424 & 0.4068 & 0.0000 & 0.3729 & 0.3729\\ 0.9032 & 0.9140 & 1.0000 & 0.1828 & 0.1828 & 0.0269 & 0.0000 & 0.4839\\ 0.6644 & 0.5753 & 0.7740 & 0.6918 & 0.5274 & 0.0000 & 1.0000 & 0.3082\\ 1.0000 & 0.4773 & 0.5758 & 0.6591 & 0.6136 & 0.2197 & 0.0000 & 0.4621 \end{array}\right]$$

Then, calculate the entropy weights *W*_*2*_ according to Eqs (11) and (12).

W_2_ = [0.0497, 0.0420, 0.0453, 0.0419, 0.3032, 0.0594,0.0400, 0.0320, 0.0611, 0.0370, 0.0300, 0.0229, 0.0209, 0.0324, 0.0327, 0.0288, 0.0636, 0.0266,0.0304]

Step 3: Determine the combined weights based on level difference maximization. The largest eigenvalue of matrix *WHW*^*T*^, *λ*_*max*_ = 0.1927, and the corresponding eigenvector is [0.7843,0.6204], that is, δ_1_ = 0.7843, δ_2_ = 0.6204. According to Eq (14), we can obtain the un-normalized combined weights and then normalize them to obtain the final combined weights, as listed in Table 5.

**Table 5:** PPP-ABS investment environment evaluation indicator weights.

Target layer	Criterion layer	Weights	Indicator layer	AHP weights	Entropy weights	Combined weights
Evaluation of PPP-ABS Investment Environment	Institutional environment	0.3168	Overall guidance	0.0143	0.0497	0.0299
			Approval process system	0.0348	0.0420	0.0379
			Information disclosure system	0.0225	0.0453	0.0325
			Tax system	0.0061	0.0419	0.0219
			Accounting system	0.0091	0.3032	0.1388
			Supervisory system	0.0531	0.0594	0.0558
	ABS market environment	0.4029	Issue size	0.0318	0.0400	0.0354
			Issue rate	0.053	0.0320	0.0437
			Settlement amount	0.0901	0.0611	0.0771
			Turnover rate	0.0901	0.0370	0.0665
			Default rate	0.1537	0.0300	0.0989
			Early repayment rate	0.0212	0.0229	0.0219
			Rating downgrade rate	0.0901	0.0209	0.0594
	Economic development environment	0.2802	GDP growth rate	0.1122	0.0324	0.0768
			Income growth rate	0.0165	0.0327	0.0236
			Fixed asset investment growth rate	0.0264	0.0288	0.0274
			Infrastructure investment growth rate	0.0396	0.0636	0.0501
			Fiscal revenue growth rate	0.0693	0.0266	0.0503
			Fiscal expenditure growth rate	0.0693	0.0304	0.0520

Subjective weights by AHP method, objective weights by entropy method, and combined weights based on level difference maximization were summarized to facilitate comparative analysis.

### 4.3 Evaluation of PPP-ABS investment environment

Step 1: Calculate the standardized score of the *i*th evaluation object on the *j*th indicator according to Eq (22) as follows:

$$Y={\left(y_{ij}\right)}_{m\times n}=\left[\begin{array}{llllllll} 0.0000 & 0.0032 & 0.0053 & 0.0096 & 0.0139 & 0.0224 & 0.0267 & 0.0299\\ 0.0000 & 0.0024 & 0.0118 & 0.0213 & 0.0213 & 0.0261 & 0.0284 & 0.0379\\ 0.0000 & 0.0046 & 0.0046 & 0.0232 & 0.0325 & 0.0325 & 0.0325 & 0.0325\\ 0.0000 & 0.0000 & 0.0219 & 0.0219 & 0.0219 & 0.0219 & 0.0219 & 0.0219\\ 0.0000 & 0.0000 & 0.0000 & 0.0000 & 0.0000 & 0.0000 & 0.0000 & 0.1388\\ 0.0000 & 0.0040 & 0.0040 & 0.0199 & 0.0518 & 0.0558 & 0.0558 & 0.0558\\ 0.0000 & 0.0034 & 0.0120 & 0.0200 & 0.0247 & 0.0322 & 0.0354 & 0.0195\\ 0.0294 & 0.0194 & 0.0437 & 0.0389 & 0.0217 & 0.0174 & 0.0118 & 0.0000\\ 0.0000 & 0.0080 & 0.0076 & 0.0160 & 0.0332 & 0.0335 & 0.0771 & 0.0705\\ 0.0000 & 0.0350 & 0.0170 & 0.0188 & 0.0331 & 0.0288 & 0.0665 & 0.0654\\ 0.0989 & 0.0791 & 0.0593 & 0.0198 & 0.0000 & 0.0593 & 0.0791 & 0.0593\\ 0.0102 & 0.0000 & 0.0180 & 0.0167 & 0.0137 & 0.0179 & 0.0196 & 0.0219\\ 0.000 & 0.0544 & 0.0370 & 0.0532 & 0.0573 & 0.0572 & 0.0593 & 0.0594\\ 0.0595 & 0.0570 & 0.0582 & 0.0558 & 0.0471 & 0.0000 & 0.0768 & 0.0099\\ 0.0209 & 0.0165 & 0.0205 & 0.0173 & 0.0146 & 0.0000 & 0.0236 & 0.0032\\ 0.0274 & 0.0200 & 0.0163 & 0.0149 & 0.0112 & 0.0000 & 0.0102 & 0.0102\\ 0.0453 & 0.0458 & 0.0501 & 0.0092 & 0.0092 & 0.0013 & 0.0000 & 0.0242\\ 0.0335 & 0.0290 & 0.0390 & 0.0348 & 0.0266 & 0.0000 & 0.0503 & 0.0155\\ 0.0520 & 0.0248 & 0.0300 & 0.0343 & 0.0319 & 0.0114 & 0.0000 & 0.0240 \end{array}\right]$$

Step 2: Determine the positive ideal solution *y*^+^and negative ideal solution *y*^−^ according to the Eqs (22) and (23).


$$y^{+}=(0.0299,0.0379,0.0325,0.0219,0.1388,0.0558,0.0354,0.0437,0.0771,0.0665,0.0989,0.0219,0.0594,0.0768,0.0236,0.0274,0.0501,0.0503,0.0520)$$

$$y^{-}=(0,0,0,0,0,0,0,0,0,0,0,0,0,0,0,0,0,0,0)$$

Step 3: Calculate the distance of each evaluation object to *y*^+^ and *y*^−^ according to Eq (24), then calculate the relative closeness of the institutional environment, ABS market environment, economic development environment, and overall investment environment from 2015 to 2022 according to Eq (25), as shown in Table 6.

**Table 6:** Relative evaluation results based on TOPSIS.

Evaluation objects	Year							
	2015	2016	2017	2018	2019	2020	2021	2022
Institutional Environment	0.0000	0.0440	0.1446	0.2328	0.3315	0.3538	0.3609	1.0000
ABS market Environment	0.4572	0.6038	0.4714	0.3257	0.3172	0.6199	0.9159	0.7787
Economic development Environment	0.8055	0.6762	0.7413	0.5931	0.5147	0.0893	0.5623	0.3122
Overall	0.4257	0.4258	0.4199	0.3828	0.3925	0.3872	0.5373	0.6528

The relative scores ((i.e., relative closeness) obtained by the TOPSIS method are listed.

**Fig 2:**
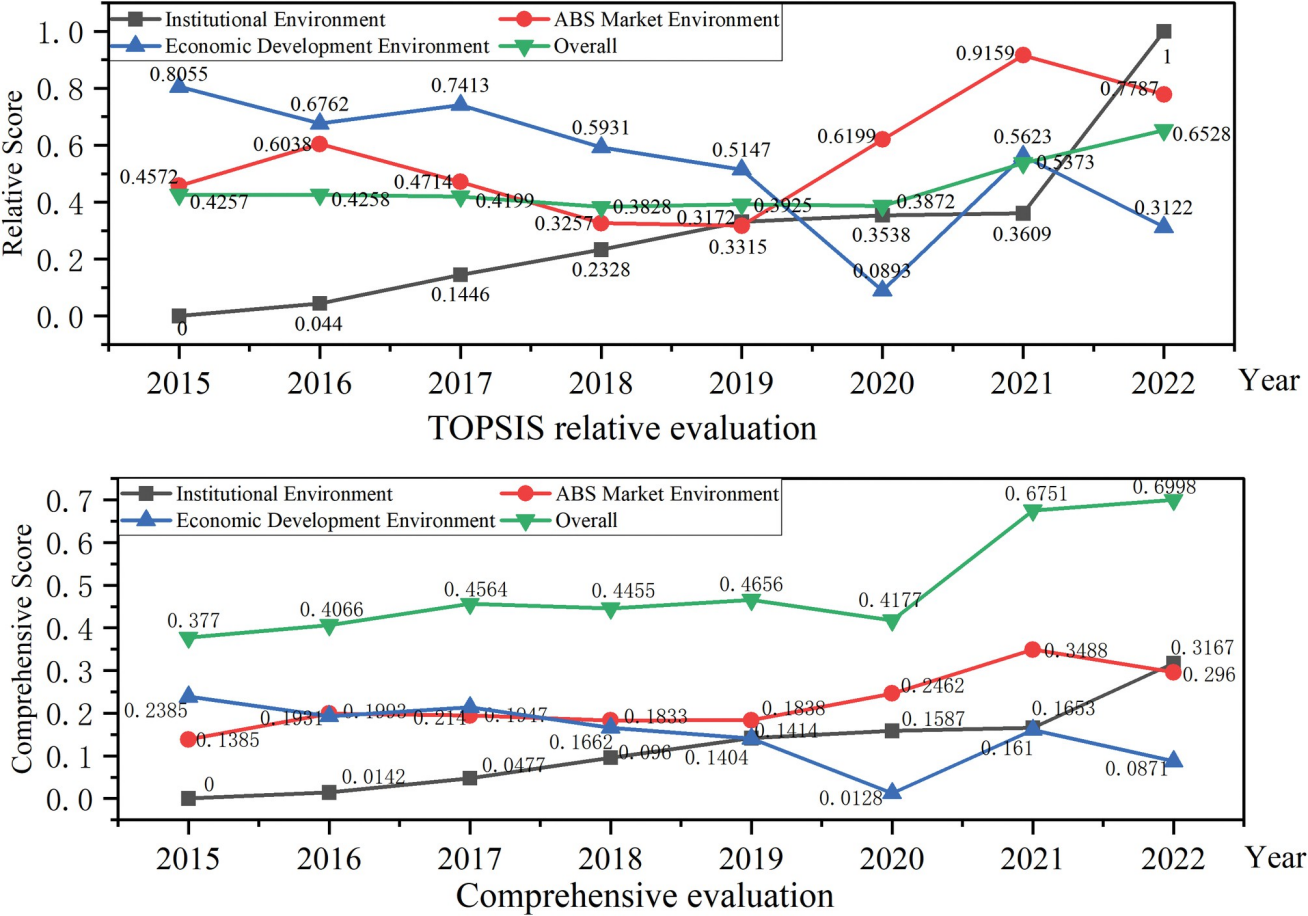
Dot-line graph of evaluation results. Visualize the changing trends of the PPP-ABS investment environment from 2015 to 2022.

**Fig 3:**
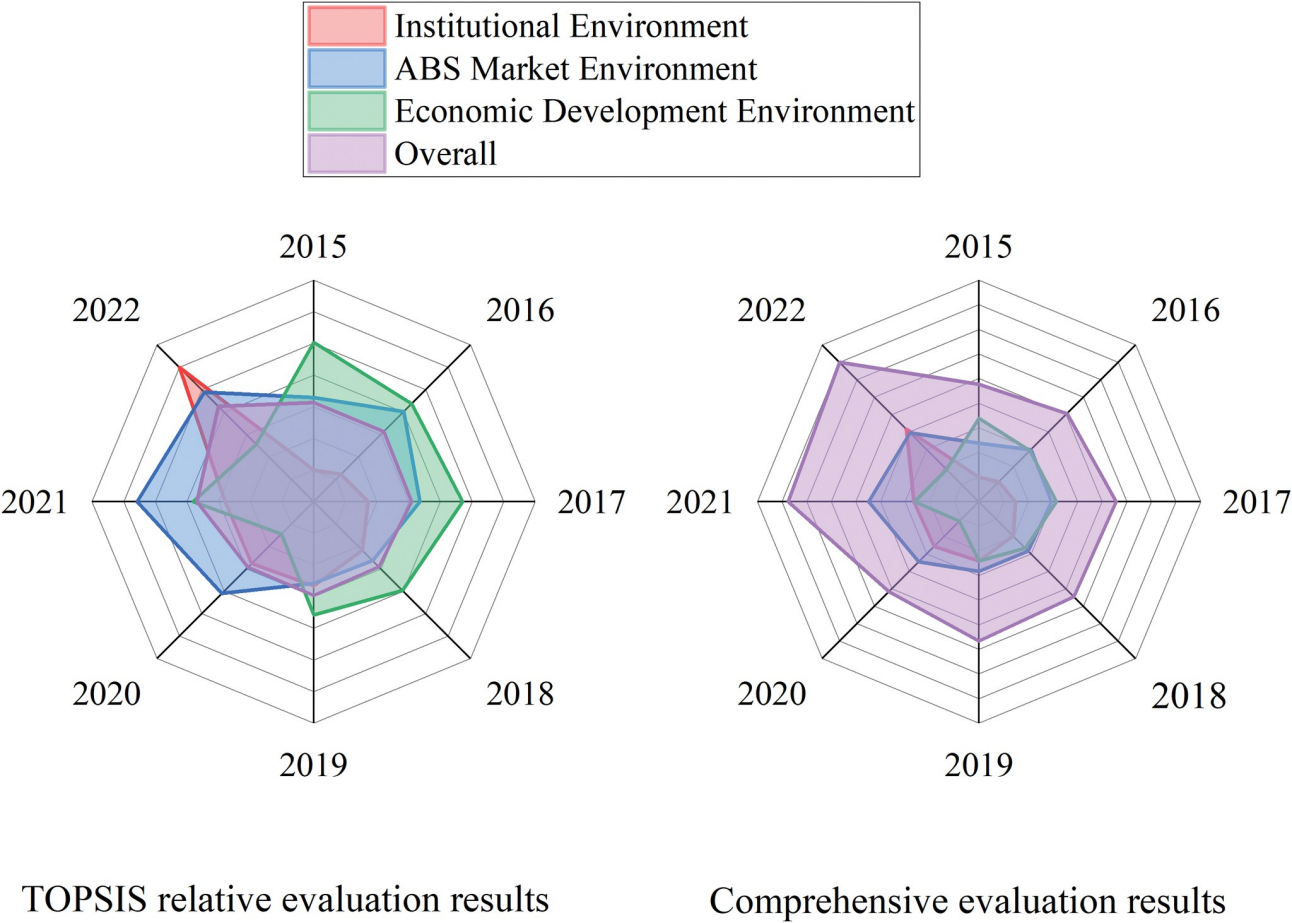
Radar map of evaluation results. Compare and display the evaluation score of each criterion level for each year more clearly.

We compared the TOPSIS evaluation results based on relative closeness with the simple weighted comprehensive evaluation results, and the results were approximately the same. The simple weighted comprehensive evaluation results are presented in Table 7. We present the results of Tables 6 and 7 in a dot-line graph and radar map to visualize the current situation of China’s PPP-ABS investment environment, as shown in Figs 2 and 3. In comparison, the TOPSIS evaluation results show the strengths and weaknesses of the PPP-ABS investment environment more clearly, and the evaluation results are consistent with the actual situation, which confirms the feasibility and effectiveness of proposed method.

**Table 7:** Simple weighted comprehensive evaluation results.

Evaluation objects	Year							
	2015	2016	2017	2018	2019	2020	2021	2022
Institutional Environment	0.0000	0.0142	0.0477	0.0960	0.1414	0.1587	0.1653	0.3167
ABS market Environment	0.1385	0.1993	0.1947	0.1833	0.1838	0.2462	0.3488	0.2960
Economic development Environment	0.2385	0.1931	0.2140	0.1662	0.1404	0.0128	0.1610	0.0871
Overall	0.3770	0.4066	0.4564	0.4455	0.4656	0.4177	0.6751	0.6998

The comprehensive scores obtained by the simple weighted comprehensive evaluation method are listed.

## 5. Results and discussion

This study evaluated the PPP-ABS investment environment based on the combined weighting of level difference maximization and TOPSIS method. Next, we separately discuss the results of the combined weights and TOPSIS evaluations.

### 5.1 The results of combined weighting method

From the combined weighting results, we can see the market environment is the most important factor for PPP-ABS investment, with a weight of 0.4029, followed by the institutional environment, with a weight of 0.3168, and the economic development environment has a relatively small impact on PPP-ABS, with a weight of 0.2802. Regarding the institutional environment, both the subjective and objective empowerment results show the relative importance of the regulation, approval process, and information disclosure systems. In terms of the ABS market environment, the annualized default rate, the settlement amount and turnover rate of secondary market have the greatest impact on PPP-ABS investment. In terms of the economic development environment, the growth rates of GDP, infrastructure investment, fiscal revenue and expenditure are relatively important. We can improve the PPP-ABS investment environment based on the weighting results by prioritizing the improvement of environmental factors with a higher weight.

### 5.2 The results of TOPSIS method

The TOPSIS evaluation results demonstrate that the overall investment environment of PPP-ABS is steadily improving, showing a steady upward trend. By analyzing the evaluation results for each criterion level, we can obtain the following conclusions.

**Fig 4:**
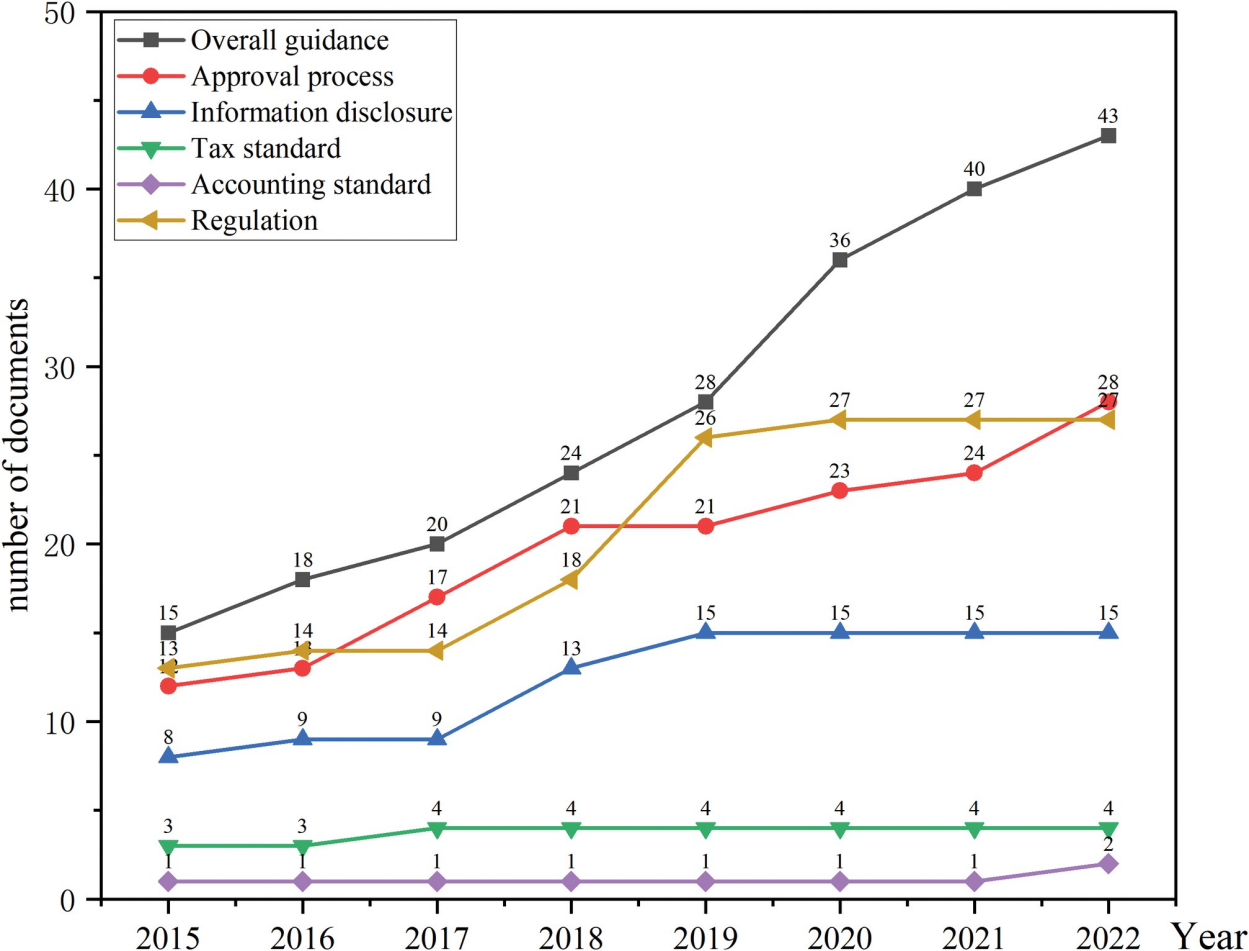
2015–2022 China’s institutional environment of PPP-ABS. The picture shows the improvement of related systems from 2015 to 2022.

The institutional environment is gradually improving. The number of documents issued by government departments indicate the importance that the government attaches to ABS, In the general environment of China’s economic transformation and upgrading, the government encourages to revitalize the stock assets, improve the efficiency of capital allocation, and serve the real economy through ABS. ABS has continuously innovated under governmental guidance. Regarding the approval process of ABS, from the beginning of the filing system to the registration system, and now to the information registration system, we can see that the approval process has been simplified, and the efficiency of issuance has been improved. From the perspective of information disclosure, the government has published various ABS information disclosure guidelines, and information disclosure has gradually become transparent and standardized. From the regulation perspective, the government attaches great importance to regulating multiple types of ABS and has published a series of regulatory measures and rules. However, accounting and tax treatment standards are relatively imperfect, and there are no special laws or regulations on ABS accounting and tax. The improvement of PPP-ABS related systems in China is shown in Fig 4.

**Fig 5:**
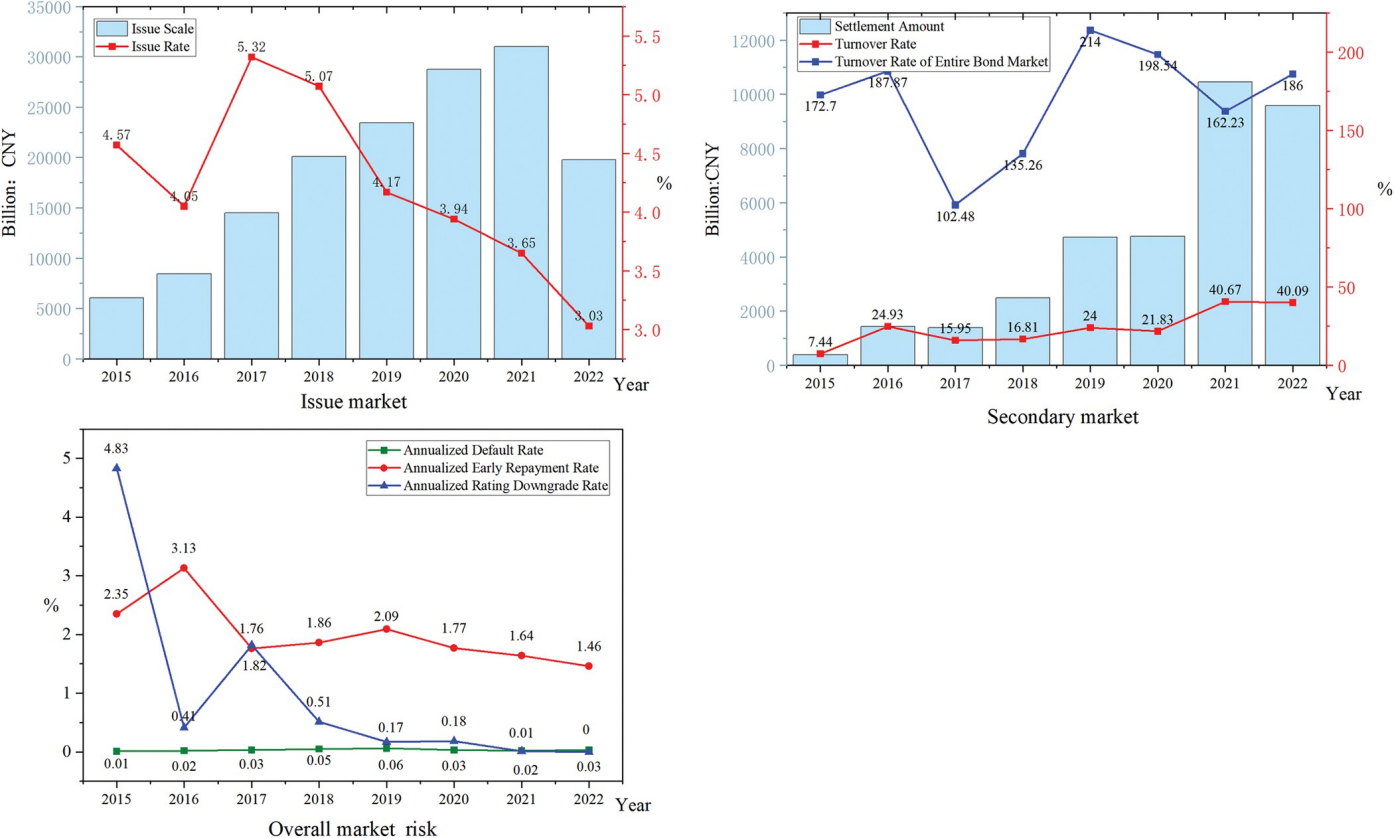
2015–2022 China’s ABS market overview. This figure illustrates the situation of the primary market and secondary market, and the overall market risk from 2015 to 2022.

The ABS market environment is generally positive. The issue size and issue rate are used to reflect the primary issue market, the settlement amount and turnover rate of the secondary market are used to reflect market liquidity, and the annualized default rate, early repayment rate, and rating downgrade rate are used to reflect the overall market risk. Regarding the issue market, the issue size continued to grow from 2015 to 2021, and the issue rate showed a downward trend after 2017. In the secondary market, the market settlement volume has continued to increase, and the turnover rate has fluctuated upward. Generally, the market liquidity has improved, but there is still a significant gap compared with the liquidity of the entire bond market. Looking at the overall risk, the annualized default rate has been maintained at a low level, and the annualized early repayment and rating downgrade rates have decreased. Therefore, the overall risk is controllable. The ABS market overview in China is shown in Fig 5.

**Fig 6:**
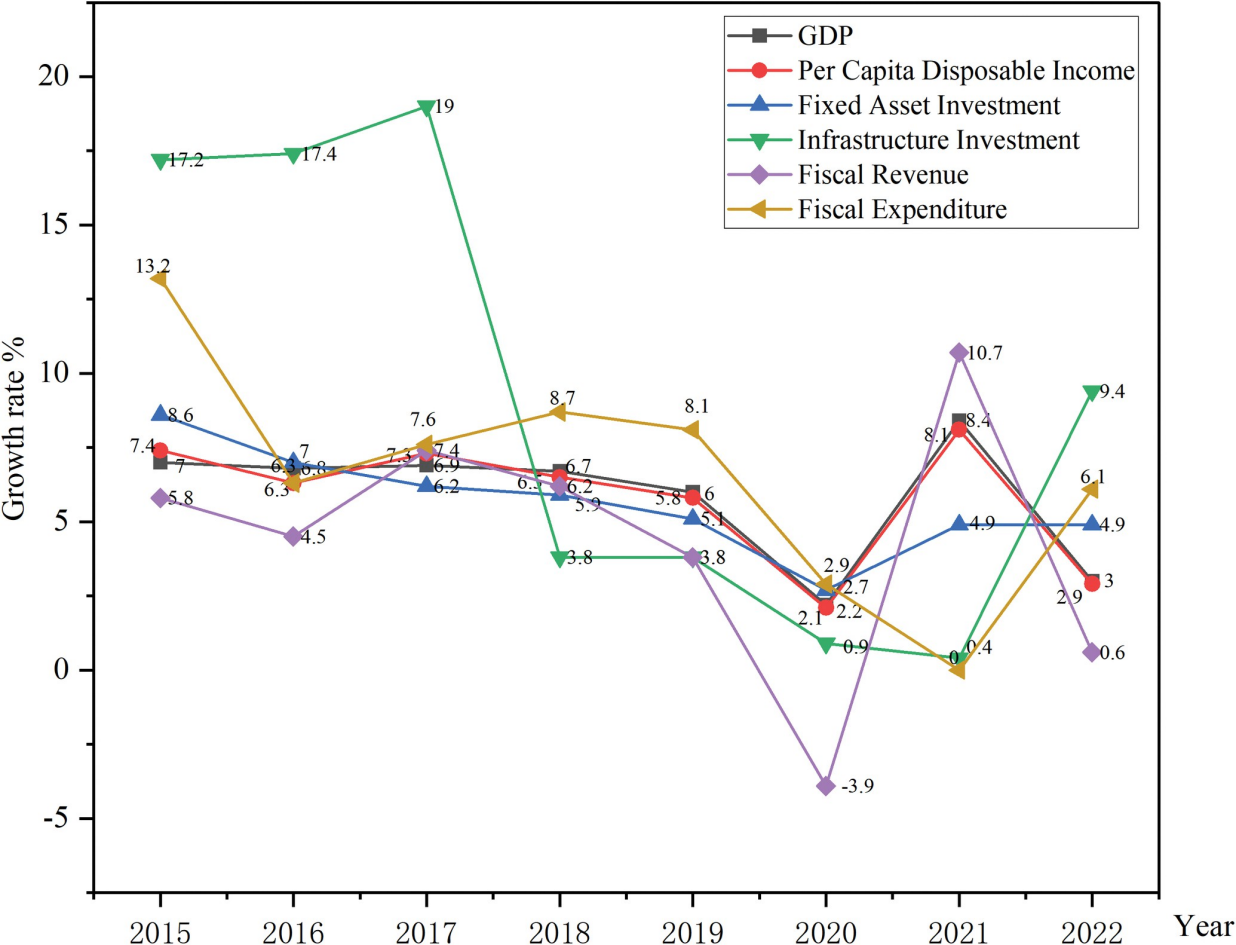
2015–2022 China’s economic development overview. This figure illustrates the changes in China’s economic development from 2015 to 2022.

The economic development environment is declining. The fluctuations of economic development indicators data are shown in Fig 6. The rate of China’s economic development gradually declined after 2015. With the COVID-19 outbreak, the economic development environment deteriorated sharply and reached a low point in 2020, with negative fiscal revenue growth. Significantly, COVID-19, as a public emergency, undoubtedly has impacted the economy. From 2019 to 2022, China adhered to the "zero" epidemic prevention and control policy, which inevitably limited economic growth. At the end of 2022, China fully liberalized its epidemic control, ending the three-year anti-epidemic era. Although the data from 2015 to 2022 show a downward trend in economic development, the economy is gradually recovering after the epidemic. The economic development environment is expected to significantly improve after 2023.

## 6. Conclusions

This study established a scientific and reasonable PPP-ABS investment environment evaluation indicator system based on the literature research and PPP-ABS practice. And the indicator weights were determined with combined weighting method based on the level difference maximization, which can compensate for the limitations of subjective and objective weighting methods and better distinguish the differences between the comprehensive evaluation results in different years. The TOPSIS method was used to evaluate the PPP-ABS investment environment from 2015 to 2022. The relative closeness obtained by TOPSIS reflects the difference between the investment environment in different years and an ideal investment environment. This study also compared the TOPSIS evaluation results with the simple weighted comprehensive evaluation results and found that they were approximately same and consistent with the actual situation, which confirms the feasibility and effectiveness of proposed method. The main conclusions are as follows:

1. The overall investment environment of PPP-ABS is steadily improving; however, some problems still need to be solved. The approval process, information disclosure, and supervisory systems are improving; however, the accounting and tax system s require further enhancement. The primary issue ABS market is relatively mature; however, the liquidity of the secondary market needs to be improved. The overall risk of the market is controllable. The economic growth slowed and showed a downward trend due to economic transformation and upgrading and the impact of COVID-19.
2. In 2016, China’s ABS market, driven by a series of favorable policies, showed a good development trend of rapid expansion and innovation, and the overall investment environment was relatively stable. Given this background, PPP-ABS was formally proposed in China. After the COVID-19 outbreak, the investment environment did not deteriorate but showed an upward trend in 2020, indicating the importance of ABS in revitalizing the stock assets and serving the real economy. The results demonstrate the rationality and feasibility of the proposal of PPP-ABS.

These results are expected to provide helpful references for institutional investors’ PPP-ABS investment decision-making. This empirical study proved that the overall investment environment of PPP-ABS is gradually improving, making it a promising investment field. These results are expected to attract securities, insurance, funds, and other institutions to invest in PPP-ABS products actively, thereby optimizing the PPP-ABS investor structure. Investors can choose appropriate PPP-ABS products based on their preferences. Active participation and support from investors will promote the sustainable and healthy development of PPP-ABS.

This study also has limitations. Due to inadequate practical experience with PPP-ABS, the evaluation indicator system cannot be perfect. In future research, we plan to improve the PPP-ABS investment environment evaluation indicator system. Additionally, this study proposes a PPP-ABS investment environment evaluation method using the combined weighting of level difference maximization and TOPSIS method, which is proven to be feasible and reliable. In the future, we will explore comparative studies based on other methods.

## Supporting information

S1 Data The AHP data. (PDF)
