# Supplementary material for: Evaluation of PPP-ABS investment environment based on combined weighting of level difference maximization and TOPSIS method
Source: PLoS One. 2023 Dec 14;18(12):e0295856. doi: 10.1371/journal.pone.0295856 (PMC10721056; doi:10.1371/journal.pone.0295856)
Supplement: S1 Data — (PDF) [file pone.0295856.s001.pdf]

The AHP data

The judgment matrix and weights of the criterion layer relative to the target layer

	Institutional environment	ABS market environment	Economic development environment	Weights
Institutional environment	1	1/3	1/3	0.14
ABS market environment	3	1	2	0.53
Economic development environment	3	1/2	1	0.33

λ_{max}=3.0536，CI=0.0268，CR=0.0516，$CR<0.1$

The judgment matrix and weights of the indicator layer relative to the criterion layer

		Overall guidance	Issue/Transaction/Registration/ Settlement	Information disclosure	Tax standard	Accounting standard	Regulation	Weights
Institutional environment	Overall guidance	1	1/3	1/2	3	2	1/4	0.10
	Issue/Transaction/Registration/ Settlement	3	1	2	5	4	1/2	0.25
	Information disclosure	2	1/2	1	4	3	1/3	0.16
	Tax standard	1/3	1/5	1/4	1	1/2	1/6	0.04
	Accounting standard	1/2	1/4	1/3	2	1	1/5	0.07

	Regulation	4	2	3	6	5	1		0.38
	λ_{max}=6.1225，CI=0.0245，CR=0.0194，CR<0.1								
ABS market environment		Issue size	Issue rate	Secondary market settlement amount	Secondary market turnover rate	Annualized default Rate	Annualized early repayment rate	Annualized rating downgrade rate	Weights
	Issue size	1	1/2	1/3	1/3	1/4	2	1/3	0.06
	Issue rate	2	1	1/2	1/2	1/3	3	1/2	0.10
	Secondary market settlement amount	3	2	1	1	1/2	4	1	0.17
	Secondary market turnover rate	3	2	1	1	1/2	4	1	0.17
	Annualized default Rate	4	3	2	2	1	5	2	0.29
	Annualized early repayment rate	1/2	1/3	1/4	1/4	1/5	1	1/4	0.04
	Annualized rating downgrade Rate	3	2	1	1	1/2	4	1	0.17
	λ_{max}=7.0711，CI=0.0118，CR=0.0087，CR<0.1								
Economic development environment		GDP growth rate	Per capital disposable income growth rate	Fixed asset investment growth rate	Infrastructure investment growth rate	Fiscal revenue growth rate	Fiscal expenditure growth rate		Weights

GDP growth rate	1	5	4	3	2	2	0.34
Per capital disposable income growth rate	1/5	1	1/2	1/3	1/4	1/4	0.05
Fixed asset investment growth rate	1/4	2	1	1/2	1/3	1/3	0.08
Infrastructure investment growth rate	1/3	3	2	1	1/2	1/2	0.12
Fiscal revenue growth rate	1/2	4	3	2	1	1	0.21
Fiscal expenditure growth rate	1/2	4	3	2	1	1	0.21

λ_{max}=6.0719，CI=0.0144，CR=0.0114，CR<0.1
